# Congenital aphallia associated with congenital urethrorectal fistula: A rare case report

**DOI:** 10.1097/MD.0000000000028878

**Published:** 2022-02-18

**Authors:** Si-Si Luo, Zhe Yang, Ning Ma, Wei-Xin Wang, Sen Chen, Qi Wu, Si-Wei Qu, Yang-Qun Li

**Affiliations:** 2th Department, Plastic Surgery Hospital, Chinese Academy of Medical Sciences and Peking Union Medical College, Beijing, China.

**Keywords:** case report, congenital aphallia, congenital urethrorectal fistula

## Abstract

**Rationale:**

Aphallia is an extremely rare congenital malformation of unknown cause, with few reports in the literature. It is usually associated with other urogenital and gastrointestinal anomalies and is believed to be a result of either the absence of a genital tubercle or chromosome polymorphism. Herein, we describe an extremely rare case of congenital aphallia with congenital urethrorectal fistula and describe our treatment for this patient.

**Patient concerns:**

An 8-year-old boy was brought to our hospital by his parents because of congenital absence of the penis. The child was male per karyotype and had excess heterochromatin on chromosome 9 (46 XY with 9 qh+). No urethral orifice was identified, and urine passed rectally since birth; thus, urinary tract outlet obstruction led to urine reflux from the anus to the epididymis for a long time. The boy had to be placed on prophylactic antibiotics because he developed urinary tract infection and epididymitis almost every day.

**Diagnosis:**

Congenital aphallia (46 XY normal male karyotype) associated with congenital urethroretal fistula.

**Interventions:**

We performed urethral exteriorization via perineal urethroplasty and urethrorectal fistula repair. The parents approved for phallic reconstruction when the boy reached puberty.

**Outcome:**

A new external urethral orifice was created on the lower scrotum. The urinary reflux was corrected, and the epididymitis symptoms disappeared. The urethral fistula was then closed. At 8 months follow up, the patient was no longer on antibiotics and had no symptoms of urinary tract infection or epididymitis.

**Conclusions:**

Compatible treatment should be adopted to address urinary tract drainage and infection. Management requires a stepwise approach to address needs as they arise. Neophalloplasty should be performed by an experienced team in early adolescence.

## 1 Introduction

Congenital aphthalia (CA) is a rare urogenital malformation. In the literature, the incidence has been reported to be 1 in 10 to 30 million live births.^[1]^ It is defined as the absence of a penis with an ectopic urethral opening, usually associated with a well-developed scrotum, bilaterally palpable and well-descended testes, and normal testosterone production in response to gonadotropin stimulation. The diagnosis is made clinically and shows the complete absence of the corpora cavernosa and corpus spongiosum with a urethral opening along the perineal midline.^[2]^ The differential diagnosis of penile agenesis includes a concealed penis, rudimentary penis, micropenis, severe hypospadias, penoscrotal transposition, male pseudohermaphroditism, or intrauterine amputation of the penis.^[3]^

Herein, we describe a case of total absence of the penis in a patient with congenital urethroretal fistula who did not have any other embryological abnormality, and we performed urethral exteriorization via perineal urethroplasty to relieve the pressure and infection. It is vitally important to understand which aspect of reconstructive surgery is important to patients and their families to properly manage their expectations. This experience reveals a possible solution to this rare congenital malformation.

## 2 Ethical statement and consent

We confirmed that all methods described in our article were performed in accordance with the relevant guidelines and regulations. The institutional review board and ethics committee of the Plastic Surgery Hospital, Chinese Academy of Medical Sciences, and Peking Union Medical College approved this ethical and methodological investigation. We obtained permission from the patient's parents to publish this rare case report, including the images.

## 3 Case Report

**Figure 1:**
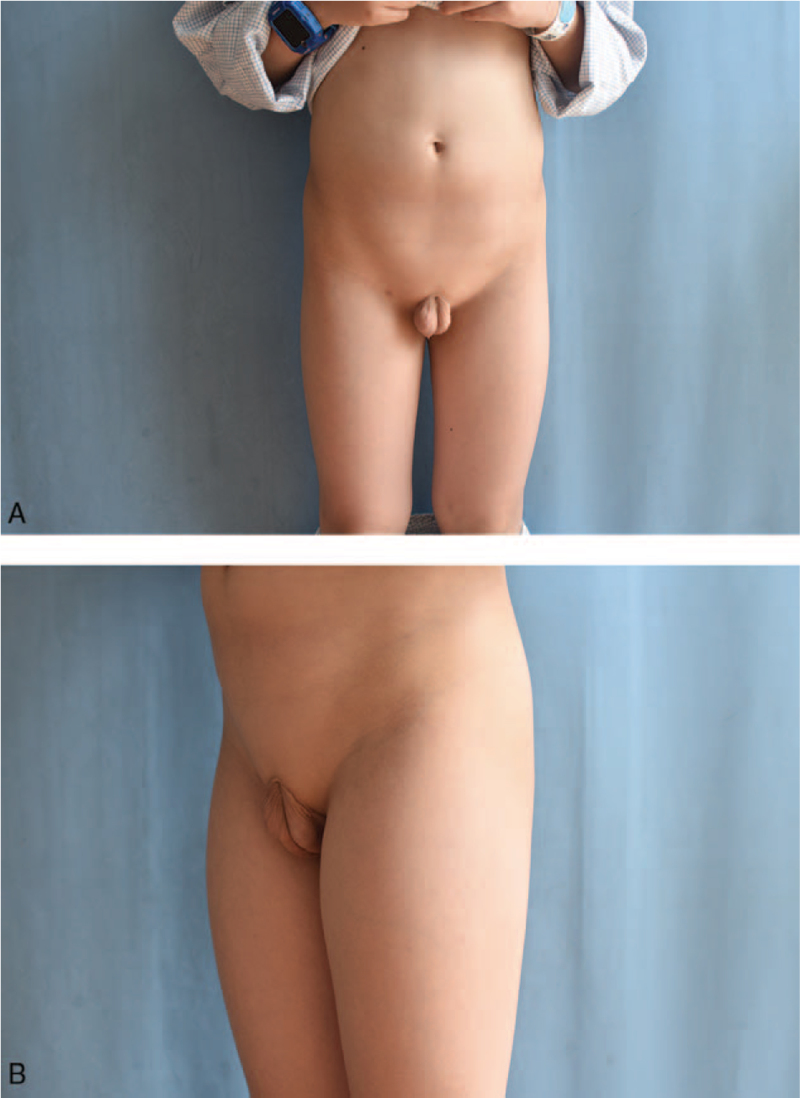
(A) Frontal (B) lateral absence of penis with well-developed scrotum and fully descended tests.

**Figure 2:**
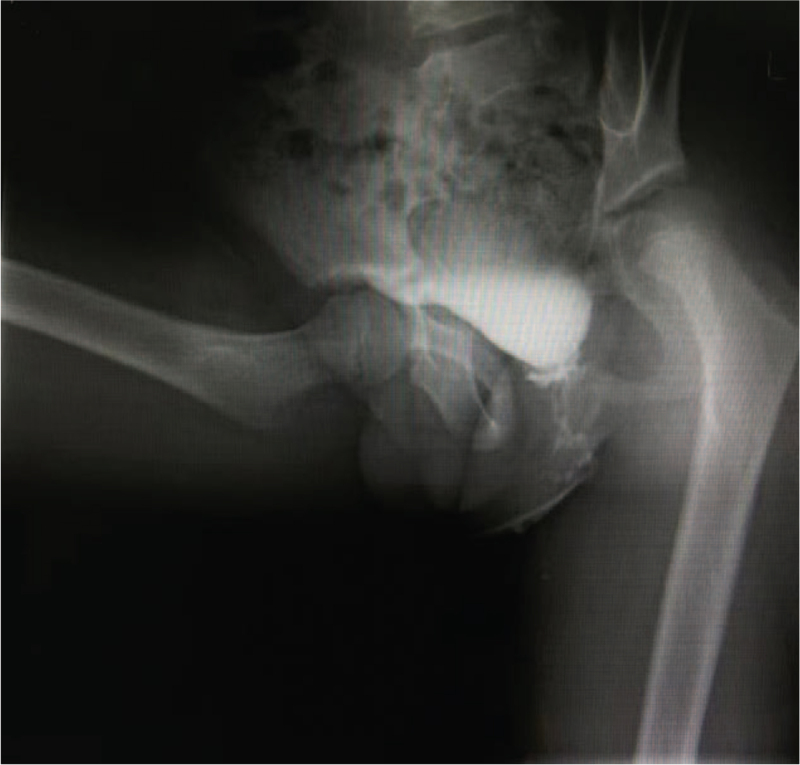
Anterograde urography demonstrating urethrorectal fistula.

**Figure 3:**
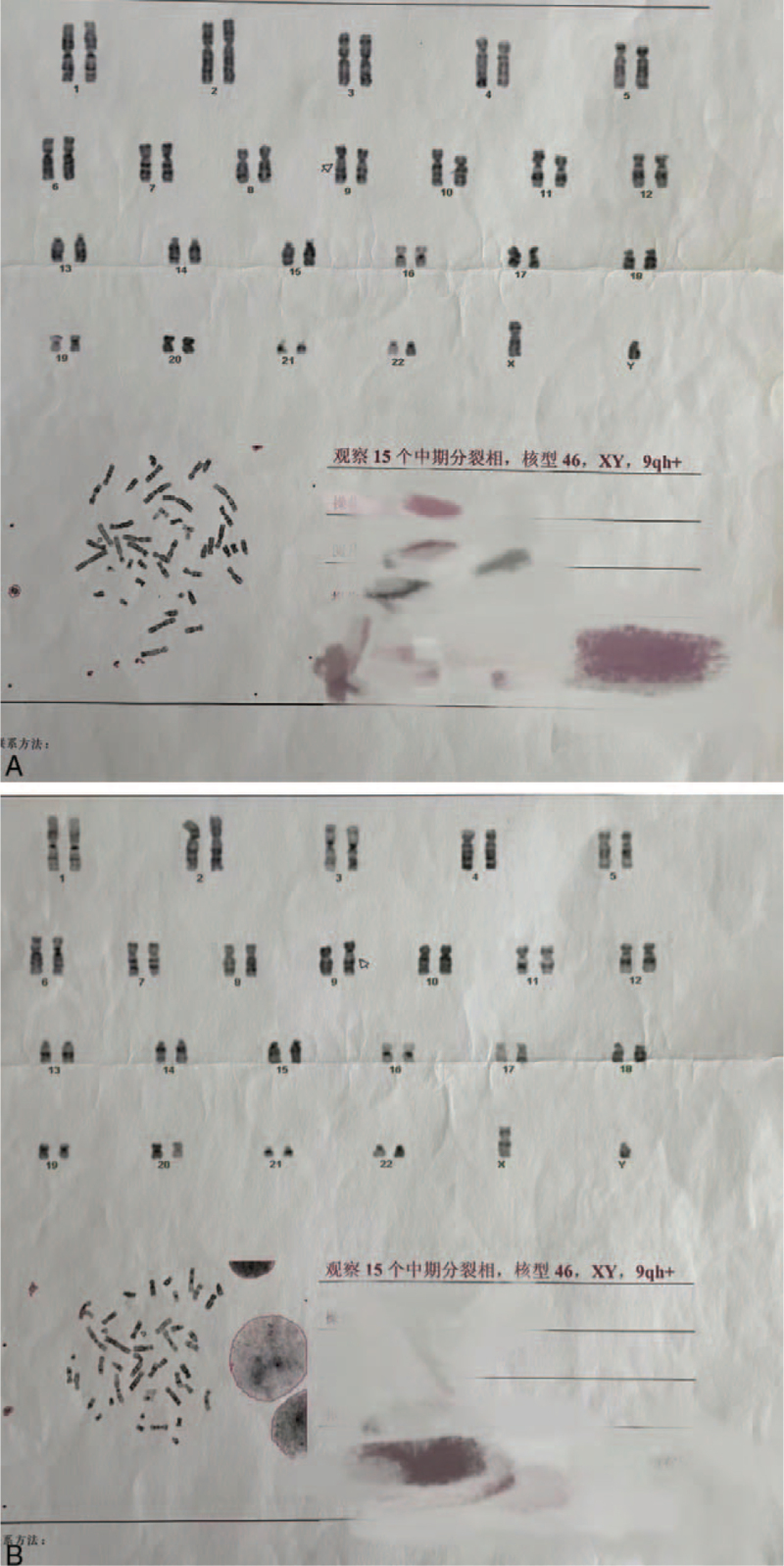
(A) Father; (B) son. Analysis of chromosomal karyotype of the patient and his father showed 46,XY,9qh+.

**Figure 4:**
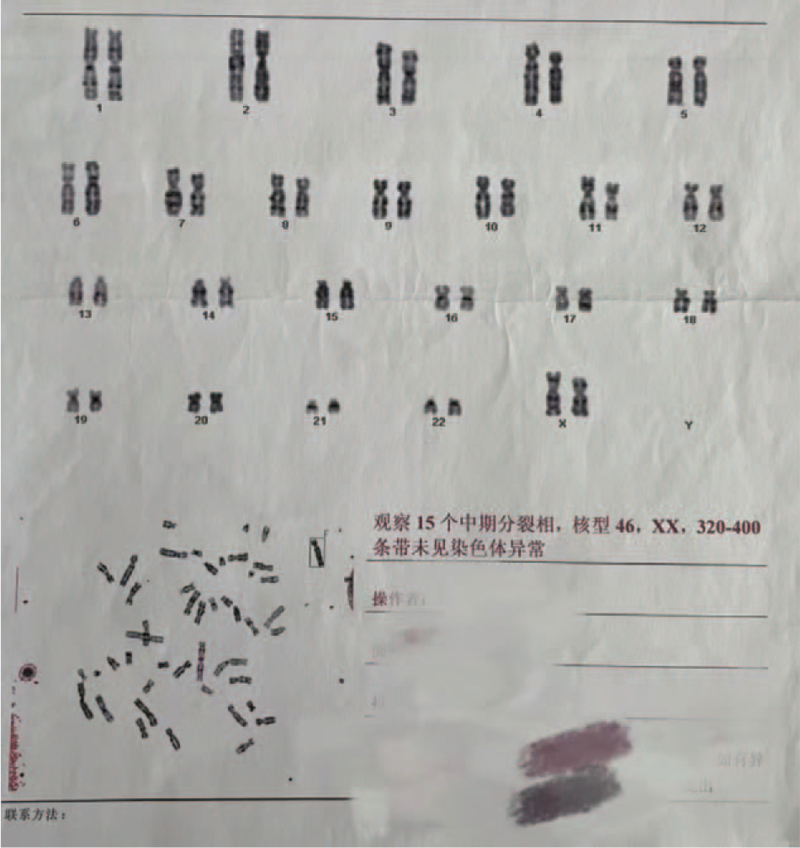
Analysis of chromosomal karyotype of mother showed 46,XX,320-400 bands without abnormality.

We report the case of an 8-year-old boy who was born without a penis and whose urine passed rectally, with urinary reflux from the anus causing long-term epididymitis. The boy had to use antibiotics to control or prevent testicular swelling and pain symptoms almost every day since he was 5 years old. Physical examination revealed absence of penis. Additionally, the scrotum appeared normal, the testes were palpable bilaterally, and no urethral orifice could be identified (Fig. 1). The patient had no other identifiable external anomalies. His blood biochemistry and routine hematologic tests were normal, urine analysis was normal, and hormonal profile was within normal limits. Echocardiography, electrocardiogram, chest computed tomography, and transabdominal ultrasound findings were normal. Anterograde urography revealed trafficking between the urethra and rectum, and contrast medium filled the rectum through a poorly visualized tract (Fig. 2). Both parents were healthy and had no family history of congenital disorders. The boy's karyotype was confirmed to be 46, XY with 9 qh+ (Fig. 3A). The father's karyotype was 46,XY with 9 qh+ (Fig. 3B). The karyotype of the mother was 46,XX (Fig. 4). The second son of this couple was a 7-year-old boy diagnosed with a concealed penis.

The greatest reconstruction challenge in patients with aphthalia is urethroplasty. We performed urethral exteriorization via perineal urethroplasty for the urinary division of the urethrorectal fistula. We fashioned scrotal skin to create a urinary tract structure as the posterior urethra. A new external orifice of the urethra, located on the lower scrotum, was created to decompress the pressure of the urinary tract and the infection. The urinary reflux was corrected, and the urethrorectal fistula was closed. The postoperative course was uneventful, and the patient was discharged 10 days postoperatively. At the 8-month follow-up, the patient had not received prophylactic antibiotics and had no symptoms of urinary tract infection or epididymitis. After discharge, the patient was able to urinate through the newly created urethral external orifice. After explaining the nature of the abnormality and management options to the parents, it is anticipated that the patient will undergo nephalloplasty at the time of puberty (age 10–15 years) when he reaches a height of at least 160 cm.

## 4 Discussion

Aphallia is a rare and serious genitourinary malformation, with fewer than 80 cases reported globally and sporadic case reports. The etiology of aphallia is unknown, but it is believed to result from either the absence of genital tubercle formation or its failure to develop during embryonic life and chromosome polymorphism. The genital tubercle forms around the fourth week of gestation by mesenchymal proliferation at the cloacal eminence. Impaired mesenchymal proliferation leads to this failure,^[4]^ and failed developmental genital tubercles are related to inadequate development of the caudal mesenchyme or an early insult in cloacal maturation.^[2]^ Aphallia is an isolated disorder in which the rest of the genitourinary tract has a normal configuration. It can also be associated with other abnormalities related to a defect in the induction of cloacal differentiation during early embryogenesis, such as a defect in blastogenesis.^[5]^ These abnormalities, including renal agenesis or dysplasia, vesicoureteral reflux, cryptorchidism, tracheoesophageal fistula, and ventricular septal defect, usually affect other systems and are present in >50% of cases (Table 1).^[^6^,^^7^^]^

**Table 1:** Genitourinary and nonurinary malformations associated with congenital aphallia.

Genitourinary malformations	Nonurinary malformations
Blind urethra	Tracheoesophageal fistula
Cystic kidneys	Ventricular septal defect
Pelvic kidneys	Annular pancreas
Vesicoureteral reflux	Imperforate anus
Abnormal renal rotation	Saddle nose
Cryptorchidism	Pigeon chest
Agenesis of the prostate	Simian creases

An increasing number of statistics show that chromosome polymorphisms are mainly reflected in the variation of heterochromatin and are associated with reproductive abnormalities. Heterochromatin refers to the portion of the genome that remains condensed and deeply stained as the cell transitions from metaphase to interphase.^[8]^ It exists in all eukaryotic chromosomes and usually lies in regions with fewer genes, such as the centromeres.^[9]^ Heterochromatin variants are believed to be clinically insignificant variations in human karyotypes, including different length patterns for heterochromatin blocks, which are marked as qh+/qh−, or pericentric inversion. Nevertheless, an increasing number of hereditary diseases or susceptibility to congenital diseases, especially urogenital malformations, have been frequently reported to be associated with these variants.^[^9^–^11^]^ Both father and sons in this case possessed 9qh+.

**Figure 5:**
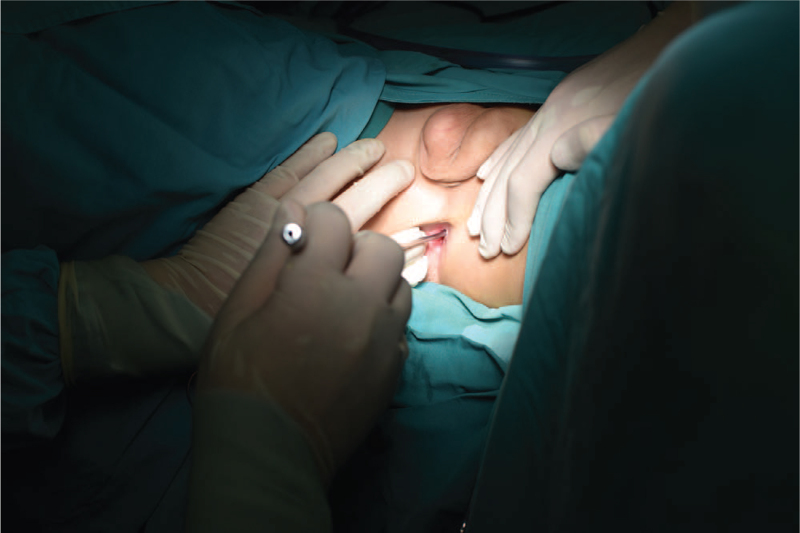
Urethral opening at anterior rectal wall, fistula above the dentate line.

Aphallia may be classified into 3 types based on the anatomical position of the ectopic urethra in relation to the anus, as described by Skoog and Belman in 1984.^[12]^ Post-sphincteric is the most common subtype (60%). Here, the urethra opens near the anterior anal verge or anywhere on the perineum in midline. It has the highest survival rate (87%) and the lowest incidence of associated malformation.^[5]^ Individuals with presphincteric aphallia account for 28% of the aphallia population, with fistulas above the dentate line and 36% mortality in the neonatal period due to life-threatening abnormalities.^[^5^,^^13^^]^ Urethral atresia, with the lowest frequency (12%), is fatal and is associated with a number of malformations.^[5]^ Our patient had the pre-sphincteric type (Fig. 5).

The following is presented in the 1970 edition of Campbell and Harrison Urology^[14]^:“We are not born with an innate attitude of identity, and how we think of ourselves is a reflection of what our parents thought we were and how we reared. Sex identity begins with the assignment of sex at birth, and in the ensuing months, the attitudes of others through daily acts create and reinforce who we are. Current thinking does not predicate the final labeling as dependent upon any one criterion of sex, and quite often, the morphology of the external genitalia will assume the most important feature and be contradictory to gonadal or genetic sex. It is well known that it is not possible to construct a serviceable penis; however, it is possible to establish a usable vagina. Adjunct surgical and endocrinologic administrations can effectively reinforce a final decision based on anatomic possibilities.” Hendren championed the conversion of patients with aphallia to a female phenotype in 1997, and for a long time, this type of management was considered easier than penile reconstruction.^[15]^ As of 2015, the recommended surgery and option was still feminizing genitoplasty for these patients; however, in infancy, bilateral orchiectomy is performed as early as possible within the first few days after birth to prevent postnatal androgen imprinting and sexual orientation, followed by vaginoplasty at the time of puberty.^[^3^,^^13^^]^ For aphallia patients who are old enough to gender identify or genetically male, they should be supported surgically as males with reconstruction of a totally functional neophallus.^[13]^

The treatment of aphallia can be staged in 3 phases: short, intermediate, and long-term.^[5]^ Short-term treatment aims to relieve urinary pressure, especially in patients suffering from urinary obstruction or infection, such as sepsis, and vesicostomy can be performed to drain the urinary tract. The intermediate term entails urethrorectal fistula division via perineal urethrostomy, and pseudophallus construction is recommended before 36 months for male psychosexual or social development.^[7]^ Long-term results of successful neophalloplasty using vascularized flaps with microsurgery around a penile prosthesis framework and urethroplasty.

Successful re-establishment of a neophallus should give the patient a male appearance outwardly, urinate standing up, and perform adequately from a sexual perspective, usually with the help of a prosthesis.

## 5 Conclusion

Congenital aphallia is usually associated with other systems, making it a major challenge, given its extreme rarity and complexity. According to the literature, the management of this malformation requires a stepwise approach to address needs as they arise, and a multidisciplinary team consisting of pediatric surgeons, plastic surgeons, and psychologists in collaboration to achieve the desired result. It is necessary to explain the nature of this disorder and the comfort of patients and their families. Early assignment of gender is necessary for the surgeon to be able to make clinical decisions and for the patient to avoid mental consequences involving gender confusion such as “gender dysphoria.” The most challenging issue is the formation of a completely functional neophallus, for which an experienced team is required to achieve a definitive result. Based on our experience, the most urgent problem should be addressed first, namely, the issue of urinary tract drainage, especially when the patient suffers from urinary tract obstruction and infection.

## Acknowledgment

The authors are grateful to the patient's parents who provided informed consent for publication.

## Author contributions

**Conceptualization:** Si-si Luo.

**Data curation:** Si-si Luo.

**Formal analysis:** Si-si Luo.

**Funding acquisition:** Si-si Luo.

**Investigation:** Si-si Luo.

**Methodology:** Si-si Luo.

**Project administration:** Si-si Luo.

**Resources:** Qi Wu.

**Software:** Qi Wu.

**Supervision:** Yang-Qun Li.

**Writing – original draft:** Si-si Luo.

**Writing – review & editing:** Zhe Yang, Ning Ma, Wei-xin Wang, Sen Chen, Si-wei Qu.
